# Recepta biopharma portfolio: clinical and pre clinical development

**DOI:** 10.1186/1753-6561-8-S4-O15

**Published:** 2014-10-01

**Authors:** M Carolina Tuma

**Affiliations:** 1 Recepta Biopharma, São Paulo, Brazil

## 

ReceptaBiopharma is a Brazilian Biotechnology company dedicated to the research and development of monoclonal antibodies and peptides as therapeutic agents for treatment of cancer.

Monoclonal antibodies (mAbs) recognize specific targets on the surface of cells, or secreted signaling molecules, and can work as anti-tumor agents by inducing tumor cell death. MAbs act by targeting the tumor cells themselves, but can also cause tumor death by targeting endothelial cells (anti-angiogenic agents) as well as cells from the immune system, such as T-cells (immunomodulators). MAbs that target tumor cells can cause tumor cell death by several processes, including induction of apoptosis by interference with survival and proliferation pathways, cytotoxicity mediated by proteins of the complement system (CDC - complement-dependent cytotoxicity) or by effector cells (ADCC-antibody dependent cell cytotoxicity), as naked agents. MAbs can also be used as carriers, conjugated with toxins or radioisotopes, leading to a broad spectrum of therapies. Over 10 mAbs have been approved as therapeutic agents for Oncology.

Before a drug can be offered to patients, there are several well-defined phases in the process of Drug Development. After discovery of a novel agent, in pre-clinical development mAbs are characterized in terms of 1) binding to the target (in vitro assays), 2) biochemical features, 3) functional activity (cell-based assays), 4) reactivity to human tissues (immunohistochemistry), 4) anti-tumor efficacy, safety and pharmacokinetics (animal studies). In order to test a novel drug in human patients, a compilation of all pre-clinical data and the design of the first clinical trial are included in a package that is submitted to the regulatory agencies. Clinical development also goes through phases: Phase I Studies evaluate safety, pharmacokinetics and dose escalation, Phase II studies evaluate safety and efficacy, Phase III Studies are designed to confirm clinical efficacy with a larger number of patients, which are critical for approval for commercialization.

Recepta´s drug development program has mAbs in discovery, in pre-clinical development and in clinical trials, as well as peptides in discovery and pre-clinical development. Recepta´s portfolio and associated challenges in drug development will be discussed.

